# Mechanical Considerations and Clinical Implications of Joint Arthroplasty Metallosis

**DOI:** 10.7759/cureus.76592

**Published:** 2024-12-29

**Authors:** Mihir Tandon, Nitin Chetla, Jordan Hodges, Akash Koul, Sohil Dharia, Dia Shah, Sai Samayamanthula, Jasraj S Raghuwanshi, Arun Sitsabeshon, Nate Oommen, Curtis J Alitz

**Affiliations:** 1 Orthopedic Surgery, Albany Medical College, Albany, USA; 2 Orthopedic Surgery, University of Virginia School of Medicine, Charlottesville, USA; 3 Orthopedic Surgery, Middlebrook Family Medicine, Middlebrook, USA

**Keywords:** metal ions, metallosis, orthopaedic complications, orthopaedic surgery, prosthetic joint failure, total joint arthroplasty

## Abstract

The treatment for osteoarthritis (OA) often requires total joint arthroplasty (TJA) when less invasive approaches fail. The annual incidence of TJA is rising. Metal-on-metal (MoM) hip and knee implants were widely used for TJA in the past, but complications have led to their decline. Many patients received MoM implants, and the complications affect many to this day. Metallosis, the accumulation of metal debris in the body, is one of the most dangerous problems associated with TJA and can cause many severe local and systemic effects*, *including inflammation, pain, and organ dysfunction.

The metal release causing metallosis is multifactorial, including wear on the articulating surfaces and trunnionosis. Key risk factors include implant design, taper topography, head size, material properties, and patient factors. Metallosis can present clinically with local pain and inflammation, and with severe systemic effects such as cardiomyopathy and neuropsychiatric symptoms. Adverse local tissue reactions and systemic cobaltism are significant concerns that necessitate early detection and intervention. Biomarkers revolving around cobalt and chromium ion levels are useful for screening and monitoring patients with signs of metallosis.

This review aims to provide primary care physicians and orthopedic surgeons with a succinct, updated understanding of the mechanisms, risk factors, and clinical implications of metallosis to better manage TJA patients. Advancements in implant materials provide opportunities to enhance future patient outcomes and reduce the incidence of metallosis, thus promoting safer and more effective TJA.

## Introduction and background

Patients with osteoarthritis (OA) who have not found relief through less invasive modes of treatment often require total joint arthroplasty (TJA). In 2014, the annual incidence of TJA in the United States was 370,770 for total hip replacements and 680,150 for total knee replacements, which is expected to increase to 635,000 and 1.26 million by 2030 [1]. Implants come in multiple configurations, including metal-on-metal (MoM). Although the use of MoM implants has decreased due to concerns about adverse reactions and high failure rates, they are still utilized in hip resurfacing procedures. This is particularly true for young and active male patients with primary OA, as hip resurfacing offers advantages such as bone conservation, reduced risk of dislocation, and straightforward revisions to total hip arthroplasty (THA), if necessary [2]. Approximately one million patients worldwide have received TJA involving MoM implants, predominantly between 2003 and 2010 [3]. Given these ongoing applications, it remains crucial to advance our understanding of metallosis.

Metallosis is primarily defined as the buildup of metal particles in the body [4]. The most commonly implicated metals are cobalt-chromium-molybdenum alloys. Cobalt-based alloys are especially suitable for TJA patients due to their enhanced high corrosion resistance, strength, and hardness, but total hip and knee replacements generate mechanical loads sufficient to pose a risk of metallosis from these materials.

The complications of metallosis develop due to the inflammatory response that arises when metal particles from an implant are released into the body [5]. These metal particles often disperse locally, causing destructive inflammatory reactions in the surrounding soft tissue and bone. Additionally, metal particles can enter the systemic circulation, leading to widespread adverse effects for the implant recipient. Locally, metallosis can cause pain, inflammation, weakness, and joint instability. Subsequently, a need for revision surgery can arise due to severe damage in the affected area. The systemic release of metal ions can impact major organ systems, leading to neuropsychiatric symptoms, cardiomyopathy, vision loss, hearing loss, nephrotoxicity, and thyroid dysfunction [6-10]. Given these serious side effects, it is imperative that clinicians fully understand metallosis to minimize potential harm to their patients.

Current reviews on this topic are complex and either take primarily a basic science or an engineering perspective. Also, many reviews miss some of the factors contributing to metallosis or lack updates from recent research [11,12]. Moreover, as this is a relatively rare and often multisystemic disease, clinicians may fail to piece together symptoms from different organ systems that stem from metallosis, leading to a delayed or missed diagnosis. This review, therefore, aims to be digestible and cover the major risk factors, the mechanisms, and the clinical presentation of metallosis, both for clinicians developing a differential diagnosis and for those in training attempting to understand this esoteric process.

## Review

Mechanisms and risk factors

The type of joint involved in the arthroplasty significantly influences the potential for metallosis. Metallosis is more common in THA than in total knee arthroplasty (TKA). Studies have found that 10% of patients with MoM TKA develop cobalturia, whereas 57% of all patients with MoM THA develop cobalturia [8]. This cobalturia has been attributed to the fact that knee replacements usually employ polyethylene as a load-bearing surface [9]. Polyethylene serves as a barrier between the metal components, reducing MoM friction and the release of metal ions. Therefore, clinicians should especially consider metallosis in the differential diagnosis for patients with a total hip replacement who exhibit the signs described later in this paper.

Metal is released from MoM implants through multiple mechanisms. One such mechanism transpires via contact between the two surfaces of the implant. A protective surface oxide film usually covers the metal surface of implants, effectively inhibiting the release of ions [10]. However, mechanical shearing from repetitive loads can disrupt this protective layer, and continued repetitive loads, in conjunction with a lack of protective film, cause the release of toxic metal ions and nanoparticles into the tissue surrounding the implants [11]. These ions and particles can cause pathology locally and can also enter the systemic circulation, potentially resulting in major organ dysfunction.

The composition of the articulating surfaces of implants influences metallosis. MoM articulation has been shown to have a higher potential for metallosis as opposed to metal on polyethylene or other non-MoM implants due to the comparatively increased friction between metal surfaces. This potential was documented through a cohort study in which an orthopaedic clinic, with patients who had cobalt-chrome hip, knee, or shoulder replacements, found that urine cobalt levels were abnormally elevated in 100% of subjects with MoM implants. Meanwhile, only 55% of subjects with metal-on-polyethylene implants exhibited cobalturia. However, given that a majority of patients with metal-on-polyethylene implants still had cobalturia, it is evident that even non-MoM articulations can lead to significant metal ion release [12]. This finding was reinforced by Isaac et al., who measured blood chromium and cobalt levels for patients with ceramic-on-metal and MoM total hip replacements over several years. MoM chromium, MoM cobalt, ceramic-on-metal cobalt, and ceramic-on-metal chromium implants were studied. All types led to progressively increased levels of the respective ions over the time course of the study. However, ceramic-on-metal implants resulted in the lowest levels, while MoM chromium implants caused the highest blood ion levels [13].

The design and mechanics of implants further impact the potential for developing metallosis. With the advent of modular head-neck junctions in THA, a new interface called the trunnion was introduced. In the context of THA, the trunnion refers to the junction between the femoral head and neck. Trunnionosis is the term used to describe the wear or corrosion of this junction. The causes of trunnionosis are multifactorial, involving fretting, chemical corrosion, and galvanic corrosion, all of which can trigger a local immune reaction [14]. Each of these three processes hosts a unique mechanism of action and can play a role in trunnionosis. Fretting is a mechanical process where continuous cyclic motions cause toggling and torsion of the trunnion, causing corrosion and metal oxidation. The mechanically assisted chemical corrosion in trunnions occurs when grooves in the trunnion lead to an insufficiency of the protective surface oxide film, in turn, causing increased corrosion [10]. Galvanic corrosion, although playing a minor role, can occur when joint implants consist of mixed alloys. When two dissimilar metals are placed in a conductive solution, as in the case of some MoM implants, mixed alloy ions can be released as one metal acts as an anode and the other acts as a cathode [15]. The trunnion, therefore, has multiple mechanisms through which it releases metal alloy ions. The overall impact is significant, as trunnions can release more ions than direct friction between MoM articulating surfaces [16].

Trunnions come in different materials and dimensions, and these factors can influence the amount of metal ions released. Nassif et al. found that among titanium-titanium, cobalt/chromium-cobalt/chromium, and cobalt-chromium/titanium head/trunnion combinations, the cobalt/chromium-titanium and cobalt/chromium-cobalt/chromium alloys exhibited greater fretting scores, indicating higher metal release [17]. Trunnion length is another important factor. A shorter trunnion length increases the risk for trunnionosis, as a shorter trunnion is associated with less flexural rigidity, and decreased rigidity causes more metal release [18].

Other aspects of implant dimensions and materials can affect the potential for metallosis. Femoral head sizes vary, with larger femoral heads offering benefits such as improved joint stability by reducing the chance of dislocation during activities like jumping [19]. Larger heads also facilitate activities of daily living (ADLs) by enhancing the range of motion and reducing the risk of neck and cup impingement. Consequently, there has been a recent shift toward using larger head sizes in THA. However, a larger femoral head increases frictional torque and trunnion stress, leading to a greater release of metal debris [20]. Surface area is another dimensional factor for metallosis in hip arthroplasty compared to TKA. The design of hip arthroplasty generates a greater surface area for erosion, macrophage metal endocytosis, and an ensuing inflammatory response [21]. Moreover, a lesser length of contact between the trunnion and the head of an implant can elevate the risk of metal release. This is due to more mechanical stress between the trunnion and the head of an implant. Greater mechanical stress across the trunnion surface increases the risk of metallosis [22]. Finally, a greater taper angle in THA will increase fretting [17].

The number of components in an implant is another consideration in metallosis. Greater modularity is achieved through an enhanced number of components, which increases the total component interface and the potential for ion release [7]. Tower et al. performed a prospective cohort study and found that hip replacements with increased modularity were at high risk for cobalturia, with 86% of patients exhibiting this condition [12]. One justification for increased modularity in hip joints is increased hip stability. Dual mobility hip implants increase stability, jump distance, and range of motion [23]. Due to these advantages, such implants are marketed to active individuals. However, the increased modularity in these implants, which typically use a cobalt-chrome liner, raises the risk for cobalturia. A separate study found elevated serum cobalt levels in 5.2% of a random sample of healthy patients with modular dual mobility total hip arthroplasties [24]. Although this percentage may seem low if even a small portion of these patients go on to develop the negative outcomes of metallosis, the disease burden may be significant.

Diagnosis and treatment

The timing of the presentation of pain and other symptoms indicative of metallosis can vary widely. Some cases have been reported as late as 26 years postoperatively, while others occur as early as six months after surgery [25]. Localized symptoms classically start with pain, instability, or sounds emanating from the implanted region. Pain and instability develop because the ions released in the surrounding tissue cause an inflammatory reaction and subsequent bone breakdown.

If metallosis becomes severe and ion levels are high enough in the blood to cause systemic effects, neuropsychiatric symptoms can develop, including mania and depression [12]. Metallic encephalopathy can manifest as altered mental status, personality changes, new-onset dementia, and Parkinsonism. Additionally, systemic metallosis can result in tremors, deafness, tinnitus, cobalt-related cardiomyopathy, hypercoagulability, and hyperthyroidism [26]. Hypersensitivity reactions with associated itching, hives, rash, fever, and fatigue can also occur. Recognizing these clinical pictures can be challenging, but in a patient with signs of joint pain and instability, allergic reactions, and organ dysfunction, metallosis should be the differential diagnosis. Imaging, C-reactive protein (CRP) levels, erythrocyte sedimentation rate (ESR) levels, cobalt levels, and chromium levels should be assessed if there is clinical suspicion of metallosis. Joint aspiration can also be performed as it may reveal a black liquid indicative of metal particles in the joint space [27].

Adverse local tissue reactions (ALTRs) are rare but significant causes of failure in hip implants. A hypothesized mechanism for ALTRs is that mechanical corrosion releases free-radical metal species, triggering cells to release cytokines, which leads to the inflammation seen in ALTRs. However, this mechanism is still poorly understood and remains a topic of debate. While ALTRs were initially thought to occur only in MoM implants, there have been increasing cases of non-MoM ALTRs, particularly in ceramic-on-ceramic THA and 36 mm metal-on-polyethylene THA. Clinically, ALTRs can present as benign, aseptic masses or bursae in the periprosthetic tissue. Histologically, ALTRs are characterized by lymphocytic infiltration, synovial surface destruction, necrosis, and fibrin exudates [28], [29].

Current biomarkers for measuring the likelihood of an ALTR occurring include [Co] > 1.5 mcg/L, [Cr] > 7 μg/L, and a Co/Cr ratio > 1.4. Clinically, patients with a low Co/Cr ratio tend to have fewer symptoms even with high cobalt and chromium concentrations. However, there is mixed evidence regarding the accuracy of the Co/Cr ratio in predicting ALTR occurrence. Blood metal ion levels can be used to screen patients undergoing MoM hip resurfacing arthroplasty, while a combination of symptomatic patient state and blood metal ion levels may be used to determine ALTR risk in MoM THA [30].

One presentation of ALTR is a pseudotumor, an autoimmune reaction resulting in a localized mass of lymphocytes, macrophages, and metallic debris [31]. Case reports of this phenomenon describe how these immune cells activate osteoclasts, leading to bone resorption. Pseudotumors can often mimic neoplasms or infections. Appropriate workup usually involves multimodal imaging such as technetium-gallium scintigraphy, X-rays, MRI, CT, and ultrasound to differentiate pseudotumors from neoplasms [32].

MRI is typically the best imaging to analyze soft tissue and signs of metallosis. However, it has traditionally been limited to detecting metallosis due to signal loss and distortion caused by metallosis [33]. Metal Artifact Reduction Sequences (MARS) in MRI is a technological advance that uses a specific algorithm that can be used to reduce this artifact [34]. In metallosis, this can help identify abnormal imaging in patients who may present with signs of metallosis but normal imaging results.

The histopathology of metallosis reveals three main signs. First, metal-laden macrophages are present, resulting from the phagocytosis of metal released from implants. Second, diffuse metal deposition occurs due to the local release of metals. Lastly, aseptic lymphocyte-dominant vasculitis-associated lesions are observed as perivascular infiltrates, which arise from the immune response around the vasculature in reaction to cobalt-chromium deposition [35].

The treatment for metallosis is dependent on patient presentation. If a patient does not have any symptoms, they are considered low risk, and conservative management with outpatient follow-up, including serial X-rays and blood chromium levels, is sufficient. However, in patients with significant localized symptoms, revision joint surgery may be necessary. Moderate-risk patients are ones with mild pain and no implant migration. These patients require six months of follow-up, with revision if symptoms, imaging abnormality, or metal ion levels increase. High-risk patients are those with severe pain, implant migration, and high metal ion levels. These patients require urgent revision [7].

Chelation therapy has been studied as a potential treatment for systemic metallosis. However, arthroplasty-associated metallosis typically causes high levels of localized metal ions, which cannot be targeted by chelation therapy alone. Therefore, revision joint surgery is the treatment of choice in patients with systemic manifestations of metallosis as such procedures can eliminate the source of the metal ions [36]. Once this source is removed, serum metal ion levels reduce and symptoms usually improve.

## Conclusions

Metallosis is a dangerous, multifactorial phenomenon that can occur in patients treated with TJA, especially those with MoM implants. Although the use of MoM implants has decreased, thousands of patients still have MoM implants that were placed before recent technological advances. Major risk factors for metallosis include the types of surface layers of a joint, the metal identities used in the implant, and the mechanical composition of the joint. Clinicians should keep all these factors in mind when evaluating a patient suspected of having metallosis. Symptoms are varied and can affect nearly every organ system, but usually begin with joint pain and instability. Treatment for patients requiring intervention is typically revision joint arthroplasty. Understanding the mechanisms of metallosis, as outlined in this paper, enables better recognition and treatment of the disease should clinicians encounter it.
